# Mental Health Consequences of COVID‐19 Vaccine Side Effects: Findings From a Cross‐Sectional Analysis

**DOI:** 10.1002/hsr2.70998

**Published:** 2025-06-30

**Authors:** Md. Shajadul Islam, Md. Bony Amin, Md. Hafizur Rahman, Fardana Binte Zaman, Sajib Rangder, Faiaz Morshed Khan Jilan, Wajihantun Nesa Chowdhury, Sanjarah Tamim, Nusrat Jahan Hridi, Md. Hasanuzzaman, Md. Shafiqul Islam Khan

**Affiliations:** ^1^ Department of Food Microbiology, Faculty of Nutrition and Food Science Patuakhali Science and Technology University Patuakhali Bangladesh; ^2^ Department of Environmental Health and Sanitation, Faculty of Nutrition and Food Science Patuakhali Science and Technology University Patuakhali Bangladesh; ^3^ International Centre for Diarrhoeal Disease Research, Bangladesh (icddr,b) Mohakhali Dhaka Bangladesh; ^4^ Interdisciplinary Institute for Food Security Bangladesh Agricultural University Mymensingh Bangladesh; ^5^ Faculty of Nutrition and Food Science Patuakhali Science and Technology University Patuakhali Bangladesh; ^6^ Department of Cellular and Molecular Biology, Faculty of Biotechnology and Genetic Engineering Chattogram Veterinary and Animal Sciences University Chattogram Bangladesh

**Keywords:** mental health, side effects, vaccine adverse effects, vaccines

## Abstract

**Background and Aims:**

The COVID‐19 pandemic has intensified pre‐existing mental health issues in Bangladesh, yet no study has specifically examined how factors related to COVID‐19 vaccination influence mental health within this context. This study aimed to examine the relationship between mental health symptoms, including depression, anxiety, and stress, and COVID‐19 vaccination status and related factors among the adult Bangladeshi population.

**Methods:**

A cross‐sectional study with a convenience sampling technique was employed to gather the data. The sample size and response rate were 1085 and 93.68%. The mean age of the participants was 34.30 (SD: 12.79), and 55.3% were female. Descriptive and inferential statistics were carried out.

**Results:**

The reliability of the instruments, measured by Cronbach's alpha, was: depression (0.78), anxiety (0.82), stress (0.83), and overall DASS (0.92). The average number of COVID‐19 vaccine doses taken was 2.33. Each additional dose of the COVID‐19 vaccine significantly reduces stress scores by 0.61. Additionally, pre‐vaccination concerns about side effects significantly increase anxiety scores by 0.63. Furthermore, experiencing pressure to get vaccinated is associated with a significant increase in depression scores by 0.78. Moreover, experiencing negative outcomes from the vaccine significantly decreases scores across all three mental health metrics: depression by −0.91, anxiety by −0.87, and stress by −1.35.

**Conclusion:**

This study reveals the broad psychological consequences of COVID‐19 vaccination in Bangladesh, which underscore the importance of effective communication and supportive strategies, alongside targeted psychological interventions at vaccination sites.

## 1 Introduction

The outbreak of COVID‐19 has posed significant challenges worldwide, affecting physical health, social structures, and economic systems [1, 2, 3, 4]. In Bangladesh, the pandemic has exacerbated the existing burden of mental disorders, with common mental health conditions such as depression, anxiety, and stress becoming more pronounced [5, 6, 7, 8]. Before the pandemic, the prevalence of common mental disorders in Bangladesh was reported at approximately 19%, indicating a substantial baseline of mental health challenges [9]. The advent of COVID‐19 has intensified these issues, driven by prolonged social isolation, economic instability, hospitalization, and widespread health anxieties [10, 11, 12].

The introduction of COVID‐19 vaccines has been a pivotal measure in controlling the spread of the virus, with over 365 million doses administered in Bangladesh by mid‐2024 [13]. However, the vaccination process has introduced its own set of psychological stressors. These include fear of post‐immunization complications of the vaccines and anxieties about the efficacy and safety of the newly developed vaccinations. Additionally, there has been considerable stress surrounding the societal pressure and, in some cases, mandates to receive the vaccine, which for many can feel like a forced choice rather than a voluntary health decision [14, 15, 16, 17, 18].

This mix of fear and perceived coercion has led to a diverse emotional outcome among the population, manifesting in varied mental health symptoms, including heightened levels of depression, anxiety, and stress postvaccination. Research from various countries has indicated an incidence of mental health issues following vaccination, primarily due to these fears and the adverse reactions experienced or anticipated [19, 20]. However, localized research specifically exploring how these mental health challenges correlate with vaccination status and related factors within the Bangladeshi population remains scarce.

Most studies in Bangladesh have explored the demographic and socioeconomic factors associated with mental health outcomes during the COVID‐19 pandemic [6, 7, 10, 21, 22]. However, no study in Bangladesh has ever explored the COVID‐19 vaccine‐related factors affecting mental health disorders among this population. Such knowledge is paramount to provide insights that could inform targeted interventions designed to mitigate these mental health symptoms associated with the vaccination process. Therefore, this study aims to bridge this knowledge gap by examining the relationship between mental health symptoms—including depression, anxiety, and stress—and COVID‐19 vaccination status and related factors among the adult Bangladeshi population. Moreover, the findings could contribute to the development of strategies to improve the mental health support systems around vaccination campaigns, not only in Bangladesh but potentially in other similar contexts globally.

## 2 Methods

### 2.1 Study Design and Participants

We conducted a cross‐sectional survey between December 3, 2023, and March 25, 2024, across all eight administrative divisions of Bangladesh. Eligible participants were adults aged 18 years or above, without disabilities such as hearing, vision, mobility, or speech impairments, and citizens of Bangladesh not currently receiving any surgical or injury‐related treatments. Women in the antepartum and postpartum period were also excluded.

### 2.2 Sampling and Settings

We collected data from a variety of locations, including households, markets, educational institutions, parks, tea stalls, railway stations, streets, and offices, using a convenience sampling and face‐to‐face data collection technique. Employing a non‐probability sampling method, we calculated the sample size with a 3% margin of error to enhance accuracy using the cross‐sectional study formula. The minimum estimated sample size was 1068, based on a 95% confidence interval and a 50% adult population proportion for the entire 165 million Bangladeshi [23, 24]. We collected 1098 responses, and after the elimination of missing and incomplete data, 1085 valid responses were retained as the final sample size. The study's response rate was 93.7%.

### 2.3 Questionnaire and Coding

Responses were collected through a pre‐structured questionnaire covering COVID‐19 vaccine status and related factors. The questionnaire also includes sociodemographic, socioeconomic, health‐related, and mental health. This was provided in the Supporting Information S1: Table 1. COVID‐19 vaccine‐related responses were collected as binary (0 = no, 1 = yes), except for the number of COVID‐19 vaccine doses, which was recorded as a continuous variable (e.g., 0, 1, 2, 3, etc.). Depression, anxiety, and stress were assessed using the Depression Anxiety and Stress Scale 21 (DASS‐21).

#### 2.3.1 DASS‐21 Scale Bangla Version

The DASS‐21 is widely used among psychological researchers to measure mental health status and has been validated in various cultural settings, including English, Bengali, French, Spanish, and Romanian‐speaking communities [25]. This scale includes 21 items divided into three subscales: the first seven items measure depression, the next seven measure anxiety, and the last seven measure stress. Responses are collected using a Likert scale from 0 (never) to 3 (almost always), and the scores range from 0 to 21, where a higher score indicates a more severe mental health condition. The overall Cronbach's alpha for the Bangla version of the scale is 0.989 [26]. In our study, Cronbach's alpha values were 0.78 for depression, 0.82 for anxiety, and 0.83 for stress, with an overall DASS‐21 scale Cronbach's alpha of 0.92.

### 2.4 Statistical Analyses

The data analysis began with data wrangling, followed by calculating descriptive statistics such as frequency, percentages, mean, median, minimum, maximum, and standard deviation. The dependent variables were created from the scale according to guidelines. The inferential Kruskal–Wallis H and Mann–Whitney U tests were applied to explore variable associations. Prior checks confirmed data skewness. Multiple linear regression models were then executed, ensuring no multicollinearity, and confirming adequate sample size for each category [27]. Multicollinearity was assessed using the Generalized Variance Inflation Factor (GVIF) with a threshold of 10. The fit of the depression model was indicated by an *F*‐test (df: 38) with a *p* value < 0.001, similarly for anxiety and stress models. Vaccination status before the COVID‐19 pandemic was not considered as a predictor for mental health outcomes as DASS‐21 measures only concurrent conditions. All tests were two‐sided and conducted with a 95% confidence interval, and *p* values less than 0.05 were considered statistically significant. Data were analyzed and visualized using IBM SPSS 27.0 and R 4.4.0.

## 3 Results

### 3.1 Sociodemographic and Socioeconomic Characteristics of the Study Population

Table 1 presents the sociodemographic and socioeconomic characteristics of the study population. Most participants were from the Dhaka division (22%), aged 18–25 years (35%), female (55.3%), Muslim (93.5%), married (62.9%), and resided in rural areas (63%). A third had an education level of honors or above (32%), and 13.4% earned below 15,000 BDT per month. Students made up 31.9% of the sample. The average household size was 4.9 members, with 96% maintaining connections with family and friends. Additionally, 19.3% reported chronic health issues, 25.1% had regular health checkups, 71.2% engaged in physical activity, and 21.5% had a non‐communicable disease. All results were provided in the Supporting Information S1: Table 1.

**Table 1:** Sociodemographic and economic characteristics of the population.

Variables	Categories	*N* (%) or (mean ± SD)
Division	Barisal	74 (6.8)
	Chattogram	190 (17.5)
	Dhaka	239 (22)
	Khulna	128 (11.8)
	Rajshahi	94 (8.7)
	Rangpur	141 (13)
	Mymensingh	132 (12.2)
	Sylhet	87 (8)
Age in years (Mean ± SD: 34.30 ± 12.79)	18–25	376 (34.7)
	26–35	284 (26.2)
	36–45	204 (18.8)
	46–55	136 (12.5)
	56–76	85 (7.8)
Gender	Female	600 (55.3)
	Male	485 (44.7)
Education	No formal education	39 (3.6)
	Primary	145 (13.4)
	Secondary	278 (25.6)
	Higher secondary	276 (25.4)
	Hons or above	347 (32)
Monthly average family income in BDT	Below 15,000	145 (13.4)
	15,000–30,000	488 (45)
	Above 30,000	452 (41.7)
Marital status	Married	683 (62.9)
	Unmarried	365 (33.6)
	Divorced/separated	15 (1.4)
	Widowed	22 (2)
Religion	Muslim	1015 (93.5)
	Hindu	65 (6)
	Others	5 (0.5)
Area	Urban	401 (37)
	Rural	684 (63)
Occupation	Government jobs	59 (5.4)
	Nongovernment jobs	69 (6.4)
	Business	139 (12.8)
	Self‐employed	91 (8.4)
	Student	346 (31.9)
	Housewife	284 (26.2)
	No job	39 (3.6)
	Others	58 (5.3)
Total household members in the family (min–max: 1–18)	—	4.90 ± 1.95	
Total children in the household (min–max: 0–6)		1.23 ± 1.13
Connected with family and friends	Yes	1039 (95.8)
Total		1085 (100)

*Note:* Others include daily wagers, rickshaw pullers, barbers, and boatmen. Among the respondents, 209 reported chronic health issues, 272 had regular health checkups, 772 engaged in physical activity, and 233 had a non‐communicable disease.

#### 3.1.1 Vaccination and Side Effects Information

Table 2 summarizes the vaccination information of the study participants. Before the COVID‐19 pandemic, 82.9% had received some form of vaccine, and 35.4% experienced post‐immunization manifestations from previous vaccinations. On average, participants received 2.33 COVID‐19 vaccine doses, with 41.9% concerned about unintended effects beforehand and 38.6% feeling pressured to get vaccinated. Postvaccination, 55.3% reported experiencing immunogenic complications.

**Table 2:** COVID‐19 vaccination and related information of the respondents.

Variables	*N* (%) or mean ± SD
Before the COVID‐19 pandemic, received any vaccine	900 (82.9)
Experienced any side effects from previous vaccinations^a^	384 (35.4)
Doses of COVID‐19 vaccine received (min‐max: 0–3)	2.33 ± 0.77
Concerned about side effects before receiving the COVID‐19 vaccine^a^	455 (41.9)
Faced pressure from anywhere to take the COVID‐19 vaccine^b^	419 (38.6)
Experienced any side effects from the COVID‐19 vaccination^a^	600 (55.3)
Total	1085 (100.0)

^a^ Headache, fever, pain, etc.

^b^ Pressure from the workplace, family, or friends.

**Figure 1:**
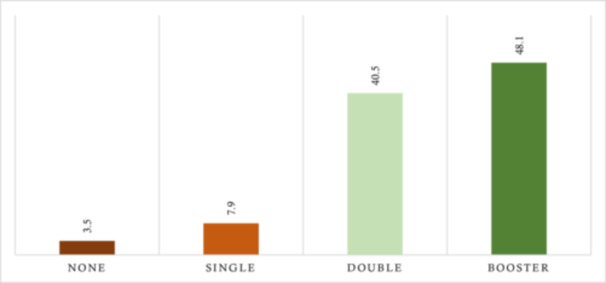
Percent distribution of participants based on the doses of COVID‐19 vaccine received (*n* = 1085). *Note:* 38 did not receive the vaccine, while 86 received a single dose, 439 completed double doses, and 522 received a booster dose.

Figure 1 shows the distribution of participants based on the number of COVID‐19 vaccines received. Nearly half (48.1%) received a booster dose, 40.5% received two doses, while only 3.5% received none.

**Figure 2:**
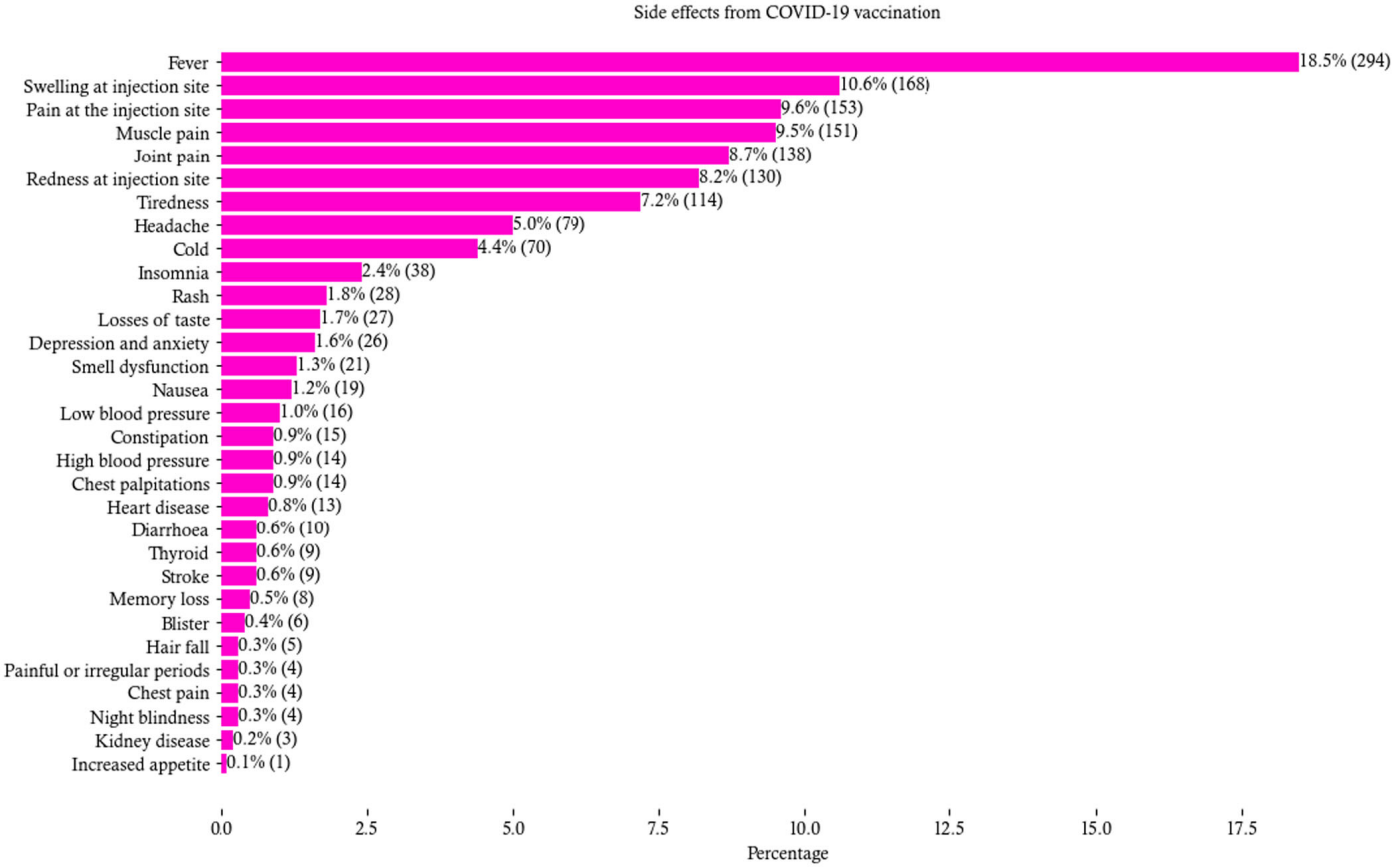
Experienced side effects from COVID‐19 vaccinations (*n* = 568). *Note:* Frequencies and percentages were derived from multiple response analysis, in which 568 respondents recorded 1591 responses, and 32 participants' data were missing.

Figure 2 depicts side effects information, experienced from the COVID‐19 vaccination. Participants reported mainly fever (19%), pain in the injection site (10%), muscle pain (10%), and joint pain (9%). Along with these, tiredness (7%), headache (5%), insomnia (2.4%), rash (1.8%), and smell dysfunction (1.3%) were notably reported vaccine‐related issues from COVID‐19 Vaccination. Besides, due to the COVID‐19 vaccinations, a tiny portion (0.3%) of the participants reported chest pain, hair fall, painful periods, night blindness, kidney disease, and increased appetite.

### 3.2 Bi‐Variate Associations of COVID‐19 Vaccination and Depression, Anxiety, and Stress

Table 3 reveals associations between vaccinations and mental health. Participants who faced pressure to get the COVID‐19 vaccine had higher depression scores (Median: 3.0, Range: 16) compared to those who did not (Median: 3.0, Range: 18). Additionally, a negative correlation was observed between the number of COVID‐19 vaccine doses received and anxiety (*r *= −0.06), and stress (*r *=−0.13). Concerned participants about adverse effects before receiving the COVID‐19 vaccine had a higher anxiety score (Median: 4.0 and Range: 16 vs. Median: 3.0 and Range: 21). Interestingly, unintended outcomes experienced participants had a lower stress score (Median: 2.0, Range: 17) compared to those who did not (Median: 4.0, Range: 21). All results were provided in the Supporting Information S1: Table 2.

**Table 3:** Bi‐variate association of COVID‐19 vaccine status and related variables and depression, anxiety, and stress (*n* = 1085).

		Depression		Anxiety		Stress	
Variables	Categories	Median (range)	Test value (*p* value)^c^	Median (range)	Test value (*p* value)^c^	Median (range)	Test value (*p* value)^c^
Doses of COVID‐19 vaccine received		*r* = −0.05 (0.053)		*r* = −0.06 (0.035)		*r* = −0.13 (< 0.001)	
Concerned about side effects before receiving the COVID‐19 vaccine^a^	No	3.0 (18)	3.66 (0.056)	3.0 (21)	7.62 (0.006)	2.5 (21)	0.02 (0.890)
	Yes	3.0 (18)		4.0 (16)		3.0 (18)	
Faced pressure from anywhere to take the COVID‐19 vaccine^b^	No	3.0 (18)	12.35 (< 0.001)	4.0 (21)	3.36 (0.067)	3.0 (21)	2.04 (0.153)
	Yes	3.0 (16)		4.0 (17)		3.0 (18)	
Experienced any side effects from the COVID‐19 vaccination^a^	No	3.0 (18)	3.43 (0.064)	4.0 (21)	3.53 (0.060)	4.0 (21)	14.01 (< 0.001)
	Yes	3.0 (18)		4.0 (20)		2.0 (17)	
Total		3.0 (18)		4.0 (21)		3.0 (21)	

^a^ Headache, fever, pain, etc.

^b^ Pressure from workplace, family, or friends; *r *= Spearman correlation coefficient.

^c^ Obtained from Mann–Whitney U test.

### 3.3 COVID‐19 Vaccination‐Related Predictors of Depression, Anxiety, and Stress

**Figure 3:**
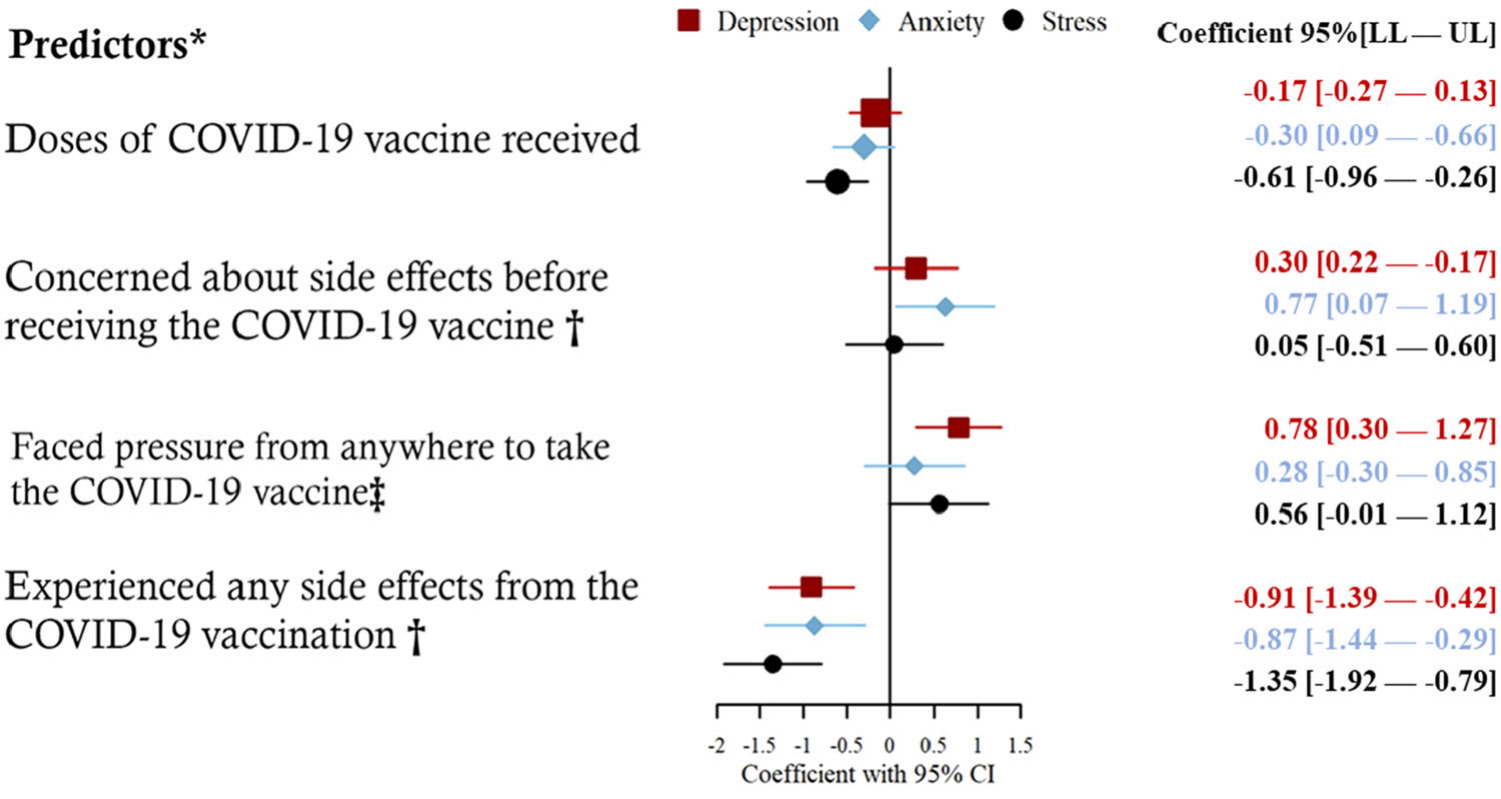
Coefficient plot of depression, anxiety, and stress. *=Adjusted for “age in years, gender, education, monthly average family income in BDT, religion, marital status, area, occupation, total household members in the family, total children in the household, connection with family and friends, presence of any chronic issue, regular health checkup, engagement in physical activity, and presence of any non‐communicable diseases”. † = Headache, fever, pain, etc., ‡ = pressure from the workplace, family, or friends.

Figure 3 shows vaccination‐related predictors of depression, anxiety, and stress. Each additional dose of the COVID‐19 vaccine significantly reduces stress scores by 0.61 (95% CI: −0.96 to −0.26). Additionally, pre‐vaccination concerns about negative outcomes significantly increase anxiety scores by 0.63 (CI: 0.07–1.19). Furthermore, experiencing pressure to get vaccinated is associated with a significant increase in depression scores by 0.78 (CI: 0.30–1.27). Moreover, experiencing side effects from the vaccine significantly decreases scores across all three mental health metrics: depression by −0.91 (CI: −1.39 to −0.42), anxiety by −0.87 (CI: −1.44 to −0.29), and stress by −1.35 (CI: −1.92 to −0.79). All results were provided in the Supporting Information S1: Table 3.

## 4 Discussion

To our knowledge, this is the first comprehensive study conducted in Bangladesh to explore the predictors of depression, anxiety, and stress associated with COVID‐19 vaccination. The relationship between COVID‐19 vaccination and mental health outcomes presents a complex interplay of psychological adjustments, societal pressures, and individual reactions to the physical experience of vaccination. Our findings delineate a nuanced portrait of these dynamics, particularly within the socio‐cultural context of Bangladesh, a nation grappling with distinct public health challenges and a diverse sociopolitical landscape.

Our study's observation that each additional dose of the COVID‐19 vaccine correlates with a significant reduction in stress (*β* = −0.61, 95% CI: −0.96 to −0.26) underscores a pivotal aspect of vaccine psychology. The alleviation of stress, particularly noted with subsequent doses, likely reflects an increasing sense of security and a cumulative reassurance regarding personal health safety. Similar patterns were reported in a European cohort study, where repeated vaccination doses were linked to decreased anxiety about COVID‐19, emphasizing the role of perceived immunity in psychological well‐being [28]. In the context of Bangladesh, where healthcare resource constraints amplify pandemic fears, the progressive reduction in stress could be attributed to heightened confidence in personal protection against the virus, thereby easing pandemic‐related existential threats.

The significant spike in anxiety associated with concerns about side effects (*β* = 0.63, 95% CI: 0.07–1.19) is reflective of a global phenomenon where vaccine hesitancy often stems from fear of postvaccination complications [29]. In Bangladesh, this is compounded by widespread misinformation and a general mistrust towards rapid medical innovations, which are not isolated cultural responses but resonate with broader hesitancy trends observed globally. For instance, a comparative study in India demonstrated a parallel increase in vaccine‐related anxiety, primarily fueled by viral misinformation on social media platforms [30]. Addressing these concerns through culturally sensitive educational campaigns that transparently discuss potential negative outcomes and their probabilities may mitigate such anxieties, fostering a more receptive attitude towards vaccination.

Our findings illustrate a significant increase in depression scores in response to perceived pressure to get vaccinated (*β* = 0.78, 95% CI: 0.30–1.27). This relationship, starkly visible in the Bangladeshi demographic, echoes similar sentiments observed in other collectivist societies like Japan, where societal expectations can heavily influence individual health behaviors [31]. The pressure to conform, potentially exacerbated by punitive measures or social ostracism for noncompliance, can engender feelings of helplessness and depression. This suggests that vaccination strategies should emphasize voluntariness and personal choice, rather than coercion, to avoid adverse mental health outcomes.

Contrary to expectations, experiencing negative outcomes led to a significant decrease in depression, anxiety, and stress scores. This intriguing outcome (depression: *β* = −0.91, 95% CI: −1.39 to −0.42; anxiety: *β* = −0.87, 95% CI: −1.44 to −0.29; stress: *β* = −1.35, 95% CI: −1.92 to −0.79) may reflect a “relief effect”. That is, the confirmation of vaccine action through physical symptoms might reassure individuals about the efficacy of the vaccine, thus alleviating broader anxieties about susceptibility to COVID‐19. This phenomenon has been less observed in Western populations, where vaccine‐attributable reactions are more likely to spur regret or fear [32], highlighting potential cultural differences in interpreting physical symptoms.

Our study has some limitations as it involves a cross‐sectional design and non‐probability sampling. In addition, responses taken on vaccinations were dependent on recall, as recall bias may occur. Furthermore, numerous factors that influence mental health might be overlooked by our study. As our study's limitation on causal inference, to get clearer understanding, further investigations are necessary, and a qualitative study could be the best approach. Despite these limitations, the study's strengths include its broad demographic representation, covering a wide range of ages, educational backgrounds, income levels, and living areas. This broad applicability enhances the generalizability, and the robustness of our adjusted predictive models increases the acceptability of these findings. However, researchers should investigate the interplay of mental health and vaccination routinely and consider employing probability sampling methods, and longitudinal designs to mitigate these limitations.

## 5 Conclusion

This study underscores the multifaceted mental health outcomes of COVID‐19 vaccination in Bangladesh, demonstrating that while additional doses reduce stress, pre‐vaccination concerns and vaccination pressures increase anxiety and depression, respectively. Interestingly, experiencing side effects tends to decrease mental health symptoms. These insights emphasize the importance of effective communication and supportive strategies to improve vaccine uptake and mental health outcomes, which are crucial for strengthening public health responses during pandemics. Additionally, our findings suggest the need for targeted psychological interventions at vaccination sites to address and mitigate the postvaccination mental health effects associated with the COVID‐19 vaccination process.

## Author Contributions

**Md. Shajadul Islam:** conceptualization, formal analysis, methodology, writing – original draft, writing – review and editing. **Md. Bony Amin:** conceptualization, methodology, supervision, formal analysis, visualization, writing – original draft, writing – review and editing. **Md. Hafizur Rahman:** visualization, writing – review and editing, methodology. **Fardana Binte Zaman:** visualization, writing – review and editing. **Sajib Rangder:** visualization, writing – review and editing. **Faiaz Morshed Khan Jilan:** investigation, writing – review and editing. **Wajihantun Nesa Chowdhury:** investigation, writing – review and editing. **Sanjarah Tamim:** investigation, writing – review and editing. **Nusrat Jahan Hridi:** investigation, writing – review and editing. **Md. Hasanuzzaman:** writing – review and editing. **Md. Shafiqul Islam Khan:** writing – review and editing.

## Ethics Statement

The authors assert that all procedures contributing to this study comply with the ethical standards of the relevant national and institutional committees on human experimentation and with the Helsinki Declaration of 1975, as revised in 2013. All procedures involving human participants were approved by the Patuakhali Science and Technology University ethical committee under approval number PSTU/IEC/2023/86.

## Consent

Participants in this study were informed, and both oral and written consent were obtained prior to data collection.

## Conflicts of Interest

The authors declare no conflicts of interest.

## Transparency Statement

The lead authors, Md. Shajadul Islam and Md. Bony Amin, affirms that this manuscript is an honest, accurate, and transparent account of the study being reported, that no important aspects of the study have been omitted; and that any discrepancies from the study as planned (and, if relevant, registered) have been explained.

## Supporting information

Supporting information.

## Data Availability

The data sets are available from the corresponding author upon reasonable request at bonyamin66bs@gmail.com.
