# Supplementary material for: Mental Health Consequences of COVID‐19 Vaccine Side Effects: Findings From a Cross‐Sectional Analysis
Source: Health Sci Rep. 2025 Jun 30;8(7):e70998. doi: 10.1002/hsr2.70998 (PMC12209322; doi:10.1002/hsr2.70998)
Supplement: Supplementary file 1 — Supporting information. [file HSR2-8-e70998-s001.pdf]

Mental Health Consequences of COVID-19 Vaccine Side Effects: Findings from a Cross-Sectional Analysis

Appendices

Supplementary Table 1. Items of the questionnaire.

Questionnaire sections	Questions or Statements
**Socio-demographic, economic, and other information**	Division, age (in years), gender, education, monthly average family income (in BDT), marital status, religion, area of residence, occupation, social connection with family and friends, total household members in the family, and total children in the family.
**Health-related information**	1) Presence of chronic health issues (health issues that usually last for 3 months or longer, e.g., AIDS, cancer, stroke, heart diseases, etc.), 2) Regular health checkups in the last 12 months, 3) Engagement in physical activity 150 minutes at least per week, and 4) Presence of any non-communicable diseases (e.g., COPD, diabetes, asthma, etc.)
**Previous vaccination information**	1) Before the COVID-19 pandemic, did you receive any vaccine? 2) Did you experience any side effects from previous vaccinations?
**COVID-19 vaccination information**	1) How many doses of the COVID-19 vaccine have you received? 2) Did you concern about side effects before receiving the COVID-19 vaccine? 3) Did you face any pressure from anywhere to take the COVID-19 vaccine? 4) Did you experience any side effects from the COVID-19 vaccination?
**DASS-21**	-

Supplementary Table 2. Bi-variate association of variables with depression, anxiety, and stress.

**Variable**	**Category**	**Median (Range)**					
		Depression	Test value (p-value)†	Anxiety	Test value (p-value)†	Stress	Test value (p-value)†
**Division**	Barisal	3.0 (11)	286.53 (<0.001)	2.0 (10)	301.54 (<0.001)	1.0 (8)	333.06 (<0.001)
	Chattogram	6.0 (18)		8.0 (21)		7.0 (18)	
	Dhaka	2.0 (13)		3.0 (15)		2.0 (12)	
	Khulna	3.0 (18)		6.0 (21)		3.0 (21)	
	Rajshahi	9.0 (10)		10.0 (11)		10.0 (12)	
	Rangpur	2.0 (16)		3.0 (17)		2.0 (21)	
	Mymensingh	1.0 (12)		1.0 (14)		0.0 (13)	
	Sylhet	1.0 (12)		1.0 (16)		1.0 (12)	
**Age in years (Mean ± SD: 34.30 ± 12.79)**	18 to 25	3.0 (18)	3.2 (0.525)	4.0 (21)	4.68 (0.322)	2.0 (21)	5.59 (0.232)
	26 to 35	3.0 (14)		4.0 (21)		3.0 (15)	
	36 to 45	3.0 (13)		3.0 (16)		3.0 (15)	
	46 to 55	4.0 (18)		5.0 (21)		3.0 (21)	
	56 to 76	3.0 (14)		4.0 (15)		3.0 (18)	
**Gender**	Female	3.0 (18)	0.84 (0.359)	4.0 (21)	0.34 (0.559)	3.0 (21)	0.15 (0.695)
	Male	3.0 (18)		4.0 (21)		3.0 (21)	
**Education**	No formal Education	7.0 (14)	34.52 (<0.001)	5.0 (21)	33.81 (<0.001)	6.0 (21)	29.95 (<0.001)
	Primary	3.0 (18)		4.0 (21)		3.0 (21)	
	Secondary	3.0 (18)		3.5 (21)		2.0 (18)	
	Higher Secondary	2.0 (13)		3.0 (15)		2.0 (14)	
	Hons or above	4.0 (18)		5.0 (17)		4.0 (21)	
**Monthly average family income in BDT**	Below 15000	2.0 (13)	16.13 (<0.001)	2.0 (21)	13.34 (0.001)	2.0 (15)	7.99 (0.018)
	15000 to 30000	3.0 (18)		4.0 (21)		2.0 (21)	
	Above 30000	4.0 (18)		4.0 (17)		3.0 (21)	
**Marital status**	Married	3.0 (18)	4.1 (0.251)	4.0 (21)	2.75 (0.431)	3.0 (21)	3.6 (0.309)
	Unmarried	3.0 (18)		4.0 (17)		3.0 (21)	
	Divorced/Separated/Widowed	2.0 (13)		2.0 (13)		2.0 (14)	
**Religion**	Muslim	3.0 (18)	10.29 (0.006)	4.0 (21)	8.96 (0.011)	3.0 (21)	9.43 (0.009)
	Hindu and other	3.0 (13)		5.0 (15)		3.0 (14)	
**Area**	Urban	3.0 (18)	0.67 (0.412)	4.0 (17)	5.46 (0.019)	2.0 (21)	2.08 (0.149)
	Rural	3.0 (18)		4.0 (21)		3.0 (21)	
**Occupation**	Government jobs	4.0 (13)	20.57 (0.004)	5.0 (15)	12.77 (0.078)	5.0 (15)	13.35 (0.064)
	Non-government jobs	5.0 (14)		6.0 (17)		4.0 (14)	
	Business	3.0 (14)		4.0 (16)		3.0 (18)	
	Self-employed	3.0 (13)		3.0 (21)		3.0 (14)	
	Student	3.0 (18)		4.0 (17)		2.0 (21)	
	Housewife	3.0 (18)		3.0 (21)		3.0 (21)	
	No job	4.0 (14)		4.0 (14)		4.0 (14)	
	Others	2.0 (18)		3.0 (21)		1.0 (15)	
**Connected with family and friends**	No	2.0 (13)	1.96 (0.161)	5.0 (15)	0 (0.992)	2.5 (15)	0.08 (0.775)
	Yes	3.0 (18)		4.0 (21)		3.0 (21)	

**Presence of chronic health issues**	No	3.0 (18)	11.23 (0.001)	4.0 (21)	9.67 (0.002)	3.0 (21)	4.14 (0.042)
	Yes	4.0 (14)		5.0 (16)		3.0 (18)	
**Regular health checkups in the last 12 months**	No	3.0 (18)	0.53 (0.466)	4.0 (21)	0.9 (0.342)	3.0 (21)	1.4 (0.237)
	Yes	3.5 (14)		5.0 (20)		3.0 (17)	
**Engagement in physical activity (150 minutes at least per week)**	No	2.0 (18)	13.32 (<0.001)	3.0 (21)	7.2 (0.007)	2.0 (21)	12 (0.001)
	Yes	3.0 (18)		4.0 (21)		3.0 (21)	
**Presence of any non-communicable diseases (NCDs)**	No	3.0 (18)	2.54 (0.111)	4.0 (21)	0.07 (0.790)	3.0 (21)	0.01 (0.917)
	Yes	4.0 (14)		4.0 (16)		3.0 (15)	

Note: Others include daily wagers, rickshaw pullers, barbers, and boatmen; Ϯ= obtained from the Maan-Whitney U test where the categories are less than 3, or Kruskal-Wallis H test where the categories are greater than 3.

Supplementary Table 3. Adjusted linear regression models of depression, anxiety, and stress.

		Depression					Anxiety					Stress				
		B	β	p-value	95% CI LL	UL	B	β	p-value	95% CI LL	UL	B	β	p-value	95% CI LL	UL
	(Constant)	7.85		0.000	5.19	10.52	9.20		0.000	6.03	12.37	9.30		0.000	6.18	12.43
**Age in years (Mean ± SD: 34.30 ± 12.79)**	18 to 25	-0.29	-0.04	0.599	-1.38	0.80	0.27	0.03	0.686	-1.03	1.56	-0.19	-0.02	0.775	-1.46	1.09
	26 to 35	-0.65	-0.08	0.180	-1.59	0.30	0.18	0.02	0.753	-0.94	1.31	-0.04	0.00	0.947	-1.15	1.07
	36 to 45	-0.77	-0.08	0.113	-1.72	0.18	-0.31	-0.03	0.594	-1.44	0.83	-0.47	-0.04	0.409	-1.59	0.65
	46 to 55	0.36	0.03	0.478	-0.63	1.35	1.07	0.08	0.076	-0.11	2.25	0.85	0.06	0.154	-0.32	2.01
	56 to 76															
**Gender**	Male	-0.15	-0.02	0.604	-0.74	0.43	0.01	0.00	0.985	-0.69	0.70	-0.34	-0.04	0.328	-1.02	0.34
	Female															
**Education**	Primary	-2.56	-0.23	0.000	-3.86	-1.26	-2.44	-0.19	0.002	-3.99	-0.89	-1.78	-0.14	0.023	-3.30	-0.25
	Secondary	-2.47	-0.29	0.000	-3.70	-1.24	-2.69	-0.26	0.000	-4.15	-1.22	-1.65	-0.16	0.025	-3.09	-0.20
	Higher Secondary	-3.10	-0.36	0.000	-4.37	-1.83	-3.95	-0.38	0.000	-5.47	-2.44	-2.71	-0.27	0.000	-4.19	-1.22
	Hons or above	-1.71	-0.21	0.009	-2.99	-0.42	-1.93	-0.20	0.014	-3.46	-0.40	-0.85	-0.09	0.270	-2.36	0.66
	No formal education															
**Monthly average family**	Below 15000 BDT	-0.99	-0.09	0.010	-1.74	-0.24	-1.52	-0.12	0.001	-2.41	-0.62	-0.86	-0.07	0.056	-1.74	0.02

**income in BDT**	15000 to 30000	-0.27	-0.04	0.279	-0.77	0.22	-0.53	-0.06	0.076	-1.12	0.06	-0.22	-0.02	0.467	-0.79	0.36
	Above 30000															
**Marital status**	Married	1.23	0.16	0.053	-0.02	2.48	1.06	0.11	0.165	-0.44	2.54	1.14	0.12	0.128	-0.33	2.61
	Unmarried	1.90	0.24	0.008	0.51	3.29	1.81	0.19	0.033	0.15	3.47	2.00	0.21	0.016	0.37	3.64
	Divorced/Widowed/Separated															
**Religion**	Muslim	-0.47	-0.03	0.300	-1.36	0.42	-0.59	-0.03	0.272	-1.65	0.47	-0.46	-0.03	0.391	-1.50	0.59
	Hindu and other															
**Area**	Rural	0.66	0.08	0.008	0.17	1.14	0.98	0.11	0.001	0.40	1.55	0.82	0.09	0.005	0.25	1.38
	Urban															
**Occupation**	Government jobs	-1.02	-0.06	0.195	-2.56	0.52	-1.13	-0.06	0.225	-2.97	0.70	-1.66	-0.09	0.072	-3.46	0.15
	Non-government jobs	-0.45	-0.03	0.552	-1.92	1.03	-0.12	-0.01	0.892	-1.88	1.63	-1.32	-0.07	0.135	-3.04	0.41
	Business	-1.38	-0.12	0.042	-2.71	-0.05	-0.78	-0.06	0.334	-2.36	0.80	-1.12	-0.08	0.161	-2.67	0.44
	Self-employed	-1.38	-0.10	0.050	-2.77	0.00	-0.63	-0.04	0.452	-2.28	1.02	-1.48	-0.09	0.074	-3.10	0.14
	Student	-2.17	-0.27	0.001	-3.49	-0.85	-1.39	-0.14	0.084	-2.96	0.18	-2.13	-0.23	0.007	-3.68	-0.59
	Housewife	-1.73	-0.20	0.009	-3.04	-0.43	-0.87	-0.09	0.274	-2.42	0.69	-1.69	-0.17	0.030	-3.22	-0.17

	Others	-1.33	-0.08	0.078	-2.81	0.15	-1.54	-0.08	0.088	-3.30	0.23	-2.16	-0.11	0.015	-3.90	-0.43
	No jobs															
**Total household members in the family (min-max: 1- 18)**		-0.34	-0.18	0.000	-0.49	-0.20	-0.45	-0.20	0.000	-0.62	-0.28	-0.44	-0.20	0.000	-0.61	-0.28
**Total Child in the household (min-max: 0 - 6)**		0.59	0.18	0.000	0.35	0.83	0.68	0.17	0.000	0.39	0.96	0.63	0.16	0.000	0.35	0.91
**Connected with family and friends**	Yes	0.20	0.01	0.726	-0.90	1.29	-0.25	-0.01	0.703	-1.55	1.05	-0.21	-0.01	0.747	-1.49	1.07
**Presence of chronic health issues**	Yes	1.07	0.11	0.004	0.34	1.80	1.83	0.16	0.000	0.96	2.71	0.95	0.08	0.031	0.09	1.81
**Regular health checkups in the last 12 months**	Yes	-0.22	-0.03	0.416	-0.77	0.32	0.27	0.03	0.417	-0.38	0.91	0.44	0.04	0.179	-0.20	1.07
**Engagement in physical activity (150 minutes at least per week)**	Yes	0.65	0.08	0.010	0.16	1.15	0.56	0.06	0.060	-0.02	1.15	0.79	0.08	0.007	0.21	1.37
**Presence of any non-communicable**	Yes	-0.39	-0.04	0.293	-1.13	0.34	-1.30	-0.12	0.004	-2.17	-0.43	-0.86	-0.08	0.050	-1.72	0.00

**diseases (NCDs)**																
**Doses of COVID-19 vaccine received**		-0.17	-0.03	0.272	-0.47	0.13	-0.30	-0.05	0.095	-0.66	0.05	-0.61	-0.11	0.001	-0.96	-0.26
**Concerned about side effects before receiving the COVID-19 vaccine †**	Yes	0.30	0.04	0.216	-0.17	0.77	0.63	0.07	0.028	0.07	1.19	0.05	0.01	0.873	-0.51	0.60
**Faced pressure from anywhere to take the COVID-19 vaccine‡**	Yes	0.78	0.10	0.001	0.30	1.27	0.28	0.03	0.345	-0.30	0.85	0.56	0.06	0.054	-0.01	1.12
**Experienced any side effects from the COVID-19 vaccination †**	Yes	-0.91	-0.12	0.000	-1.39	-0.42	-0.87	-0.10	0.003	-1.44	-0.29	-1.35	-0.15	0.000	-1.92	-0.79

Note: † = Headache, fever, pain, etc., ‡= pressure from working place, family, or friends., Others include daily wagers, rickshaw pullers, barbers, and boatmen.
